# Norepinephrine combined with phenylephrine versus norepinephrine in patients with septic shock: a retrospective cohort study

**DOI:** 10.1186/s12879-023-08142-x

**Published:** 2023-04-07

**Authors:** Dan He, Hai Hu, Liang Hong, Luming Zhang, Xuehao Lu, Wan-jie Gu, Jun Lyu, Haiyan Yin

**Affiliations:** 1 Department of Intensive Care Unit, The First Affiliated Hospital of Jinan University, Guangzhou, China; 2 Department of Anesthesiology, Hengyang Maternal and Child Health Hospital, Hengyang, China; 3 Department of Clinical Research, The First Affiliated Hospital of Jinan University, Guangzhou, China; 4 Department of Intensive Care Unit, Nanjing First Hospital, Nanjing Medical University, Nanjing, China; 5 Guangdong Provincial Key Laboratory of Traditional Chinese Medicine Informatization, Guangzhou, China

**Keywords:** Phenylephrine, Norepinephrine, Sepsis, Septic shock, MIMIC-III, Propensity score matching

## Abstract

**Background:**

Phenylephrine (PE) and norepinephrine (NE) may be used to maintain adequate blood pressure and tissue perfusion in patients with septic shock, but the effect of NE combined with PE (NE-PE) on mortality remains unclear. We hypothesized that NE-PE would not inferior to NE alone for all-cause hospital mortality in patients with septic shock.

**Methods:**

This single-center, retrospective cohort study included adult patients with septic shock. According to the infusion type, patients were divided into the NE-PE or NE group. Multivariate logistic regression, propensity score matching and doubly robust estimation were used to analyze the differences between groups. The primary outcome was the all-cause hospital mortality rate after NE-PE or NE infusion.

**Results:**

Among 1, 747 included patients, 1, 055 received NE and 692 received NE-PE. For the primary outcome, the hospital mortality rate was higher in patients who received NE-PE than in those who received NE (49.7% vs. 34.5%, *p* < 0.001), and NE-PE was independently associated with higher hospital mortality (odds ratio = 1.76, 95% confidence interval = 1.36–2.28, *p* < 0.001). Regarding secondary outcomes, patients in the NE-PE group had longer lengths of stay in ICU and hospitals. Patients in the NE-PE group also received mechanical ventilation for longer durations.

**Conclusions:**

NE combined with PE was inferior to NE alone in patients with septic shock, and it was associated with a higher hospital mortality rate.

## Introduction

Septic shock is a severe consequence of infection that typically has an extremely high mortality rate of 35–40% [1, 2]. Due to its clinical urgency, immediate treatment and resuscitation are required. Fluid resuscitation and vasoactive medication therapy are two important components of septic shock resuscitation [3]. Norepinephrine (NE) was recommended as the first selective vasopressor for septic shock [4], but there is increasing evidence that excessive dosing or duration of NE infusion can adversely affect patient outcomes due to its multiple effects on immunity, metabolism, and coagulation [5–7]. The Surviving Sepsis Campaign(SSC) guidelines therefore recommended adding other vasoactive drugs to decrease the adverse effects from NE [4, 8].

As a pure α-adrenergic agonist, phenylephrine (PE) has been suggested as potentially beneficial in achieving heart rate (HR) control [9, 10], and for reversing hemodynamic and metabolic abnormalities [11, 12]. However, it has also been found that PE has the potential to induce splanchnic vasoconstriction [13], decreased cerebral perfusion [14], and even an increased mortality risk [15, 16].

Currently, the effects of PE in treating septic shock are uncertain. We therefore hypothesized that the combined use of NE and PE has a similar effect to NE on patients with septic shock. We therefore conducted retrospective cohort research to compare the effectiveness of NE-PE with that of NE alone on hospital mortality and on other secondary outcomes in septic shock.

## Methods

### Data source and study populations

This study used the Medical Information Mart for Intensive Care-III (MIMIC-III) database, which is a freely available large database that contains deidentified data information of 46,476 patients who were admitted to the intensive care unit (ICU) of the Beth Israel Deaconess Medical Center between 2001 and 2012 [17, 18]. The true identity information of all patients is hidden in this database, and so informed consent was therefore not required from the patients. The author passed the relevant course training and obtained a database access certificate (number: 47907567).

The study cohort was constructed using the STROBE checklist [19]. The study was designed to compare the prognosis and outcomes of continuous infusions of NE-PE versus NE in ICU patients with septic shock based on the definition by Angus et al. [3] using the diagnostic codes of the ninth revision of ICD (78,552). The inclusion and exclusion criteria were as follows: only first ICU admissions; only patients treated with NE or NE-PE; only lengths of stay (LOSs) in an ICU exceeding 24 h; and only adult patients. Finally, 1, 747 patients were selected for the study cohort: 1, 055 patients in the NE group and 692 in the NE-PE group.

### Data extraction

Data were extracted using Structured Query Language [20], NE exposure was defined as the continuous intravenous infusion of NE during the ICU admission, and NE-PE exposure was defined as the continuous intravenous infusion of NE combined with PE. The variables included demographic information, vital signs, comorbidities, the score for disease severity, laboratory tests, medications, and other information about the patients. Basic information included age, sex, weight, ethnicity, admission type, and first care unit. Vital signs were the mean values on the first day of the ICU which included HR, mean blood pressure (MBP), respiratory rate (RR), temperature, and SpO2. The score for disease severity included the SOFA score [21], APSIII [22], and Elixhauser Comorbidity Index [23]. Comorbidities included congestive heart failure (CHF), cardiac arrhythmias, pulmonary circulation disorder, peripheral vascular disorder, neurological diseases, chronic pulmonary disease, hypertension, diabetes, renal failure, liver disease, solid tumor without metastasis, metastatic cancer, fluid and electrolyte disorders, drug abuse, and alcohol abuse. Laboratory tests on the first day of ICU admission included white blood cells (WBCs), hemoglobin, platelets, bilirubin, serum creatinine, urea nitrogen, glucose, lactate, bicarbonate, international normalized ratio (INR), potassium, and sodium. Medications included the use of vasopressin, dobutamine, and epinephrine. Other information included firstday urine output, firstday mechanical ventilation (MV), firstday renal replacement therapy (RRT), microorganism, and vasopressor use during ICU stays.

### Primary and secondary outcomes

The primary outcomes were the all-cause hospital and ICU mortality rate. Hospital mortality and ICU mortality were defined as death during hospitalization and ICU, respectively. Secondary outcomes included ICU LOS and hospital LOS, and MV duration. Acute kidney injury (AKI) at 48 h and 7 days were defined as whether AKI had occurred at 48 h or 7 days after ICU admission.

### Statistical analysis

The proportion of missing values for each variable did not exceed 20%. Variables with missing data were estimated and filled using the multiple imputation method [24]. Continuous parameters were presented as mean ± standard-deviation or median and interquartile range (25%–75%) values. Continuous variables were analyzed using variance or nonparametric tests. Categorical variables were analyzed using chi-square tests. Odds ratios (ORs) and 95% confidence intervals (CIs) were used to analyze outcomes between groups. SPSS software (version 27.0) and R software (version 4.1.3) were used for the statistical analyses.

Univariate and multivariate logistic regression model were established to assess the independent association of exposure with the primary endpoint. Furthermore, to ensure the stability of the results for the primary and secondary outcomes, propensity score matching (PSM) was used to balance the confounding factors between groups. The following variables were included in the PSM analysis: age, gender, weight, ethnicity, admission type, first care unit, HR, MBP, RR, temperature, SpO2, SOFA score, APSIII, Elixhauser Comorbidity Index, CHF, cardiac arrhythmias, peripheral vascular disorder, chronic pulmonary disease, pulmonary circulation disorders, hypertension, diabetes, renal failure, liver disease, solid tumor without metastasis, metastatic cancer, fluid and electrolyte disorders, drug abuse, alcohol abuse, WBCs, hemoglobin, hematocrit, platelets, bilirubin, serum creatinine, urea nitrogen, glucose, lactate, bicarbonate, INR, potassium, sodium, vasopressin, dobutamine, dopamine and epinephrine, microorganism, first-day urine output, firstday MV, firstday RRT, and initial NE dose. A 1:1 ratio was applied to matching using a 0.1 caliper [25]. The standardized mean difference (SMD) was calculated before and after matching to assess the differences between the two groups. When the SMD of a variable is less than 0.1, it can be considered that balance was obtained between the groups [26].

### Sensitivity analysis

Several sensitivity analyses were used to estimate the robustness of the results in two different models: PSM cohort with multivariate logistic regression model and inverse probability weighting with multivariate logistic regression model (doubly robust model). We also performed a sensitivity analysis to compare the mortality risk for ICU mortality between NE and NE-PE groups.

### Subgroup analysis

Subgroup analyses were implemented to further investigate the mortality rate between PSM groups. We included six subgroups: age (≥ 65 or < 65 years), gender, SOFA score (≥ 8 or < 8), HR (≥ 100 or < 100 bpm), CHF, arrhythmias, hypertension, diabetes, renal failure, and liver disease.

## Results

### Baseline characteristics

**Fig. 1:**
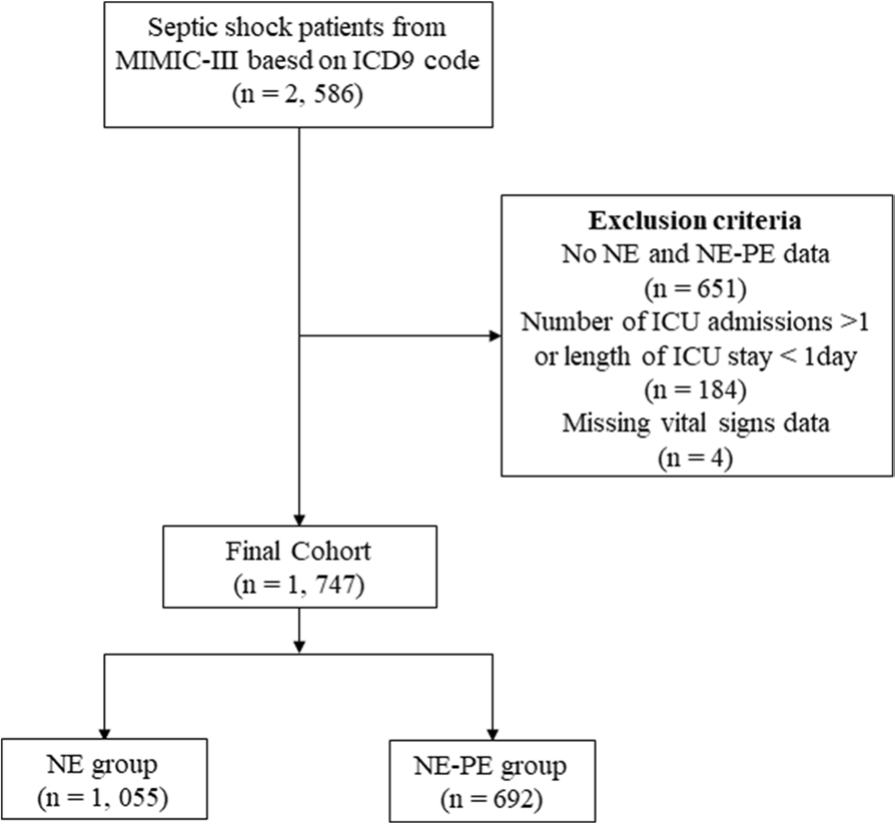
Flow diagram for patients included in the study

As shown in Fig. 1, 1, 747 patients diagnosed with septic shock [3] in the MIMIC-III database were included, comprising 1055 NE users (60.4%) and 692 NE-PE users (39.6%). The basic characteristics of the cohort are listed in Table 1. Patients who were exposed to NE-PE generally differed from those exposed to NE in most aspects during hospitalization. The NE patients had a higher proportion of Emergency admissions (97.7% vs 94.4%), MICU admissions (78.3% vs 67.9%), hypertension (53.6% vs 51.6%). The NE-PE group also had a higher percentage of CHF (43.6% vs 37.7%), cardiac arrhythmias (53.3% vs 36.4%), pulmonary circulation disorder (10.3% vs 7.3%), liver disease (28.5% vs 20.5%), fluid and electrolyte disorders (62.3% vs 56.7%). NE-PE group presented with higher severity scores after ICU admission: APSIII (75.05 ± 24.75 vs 63.45 ± 22.95), SOFA score (9.49 ± 3.94 vs 8.35 ± 3.39), and Elixhauser Comorbidity Index (14.95 ± 8.85 vs 12.63 ± 8.60). Patients in the NE-PE group also had higher HRs (98.16 ± 18.27 vs 89.40 ± 16.81 bpm) and RRs (22.26 ± 4.76 vs 21.10 ± 4.50 breaths/min). More NE-PE patients received MV (73.6% vs 56.0%) during the first 24 h of their ICU stay, and a higher proportion of NE-PE patients received other vasoactive drugs treatment during their ICU stay: vasopressin (60.3% vs 20.9%), dobutamine (9.4% vs 4.6%), dopamine (20.2% vs 16.2%), and epinephrine (7.2% vs 0.7%).

**Table 1:** Baseline characteristics of NE and NE-PE group for original cohort

Covariate	NE (*n* = 1, 055)	NE-PE (*n* = 692)	*P*-value	SMD	Missing data(%)
Age, y	67.45 (15.82)	67.11 (15.26)	0.654	0.022	0.0
Gender, female, n (%)	495 (46.9)	303 (43.8)	0.216	0.063	0.0
Weight, kg	81.62 (25.96)	83.94 (24.98)	0.064	0.093	0.1
Ethnicity, n (%)			0.264		0.0
White	781 (74.0)	503 (72.7)		0.030	
Black	109 (10.3)	59 (8.5)		0.065	
Other	165 (15.6)	130 (18.8)		0.081	
Admission type, n (%)			0.001		0.0
Emergency	1031 (97.7)	653 (94.4)		0.146	
Urgent	12 (1.1)	15 (2.2)		0.071	
Elective	12 (1.1)	24 (3.5)		0.128	
First careunit, n (%)			< 0.001		0.0
MICU	826 (78.3)	470 (67.9)		0.222	
SICU	85 (8.1)	97 (14.0)		0.172	
Other	144 (13.6)	125 (18.1)		0.115	
**Vital signs**					
Heart rate, bmp	89.40 (16.81)	98.16 (18.27)	< 0.001	0.479	0.0
Mean BP, mmHg	71.10 (7.63)	71.18 (8.69)	0.849	0.009	0.0
Respiratory rate, bmp	21.10 (4.50)	22.26 (4.76)	< 0.001	0.244	0.0
SpO2, %	96.93 (2.85)	96.24 (3.76)	< 0.001	0.183	0.1
Temperature, °C	36.81 (0.77)	36.86 (0.85)	0.168	0.064	0.4
SOFA score	8.35 (3.39)	9.49 (3.94)	< 0.001	0.299	0.0
APS III	63.45 (22.95)	75.05 (24.75)	< 0.001	0.469	0.0
Elixhauser comorbidity score	12.63 (8.60)	14.95 (8.85)	< 0.001	0.262	0.0
**Comorbidity**					
Congestive heart failure, n (%)	398 (37.7)	302 (43.6)	0.016	0.120	0.0
Cardiac arrhythmias, n (%)	384 (36.4)	369 (53.3)	< 0.001	0.340	0.0
Pulmonary circulation disorder, n (%)	77 (7.3)	71 (10.3)	0.037	0.098	0.0
Peripheral vascular disorder, n (%)	80 (7.6)	62 (9.0)	0.347	0.048	0.0
Neurological diseases, n (%)	158 (15.0)	97 (14.0)	0.627	0.028	0.0
Chronic pulmonary disease, n (%)	253 (24.0)	186 (26.9)	0.190	0.065	0.0
Hypertension, n (%)	565 (53.6)	357 (51.6)	0.450	0.039	0.0
Diabetes, n (%)	346 (32.8)	224 (32.4)	0.894	0.090	0.0
Renal failure, n (%)	250 (23.7)	177 (25.6)	0.402	0.043	0.0
Liver disease, n (%)	216 (20.5)	197 (28.5)	< 0.001	0.177	0.0
Metastatic cancer, n (%)	75 (7.1)	50 (7.2)	1.000	0.005	0.0
Solid tumor without metastasis, n (%)	46 (4.4)	31 (4.5)	1.000	0.006	0.0
Fluid and electrolyte disorders, n (%)	598 (56.7)	431 (62.3)	0.023	0.116	0.0
Drug abuse, n (%)	44 (4.2)	25 (3.6)	0.646	0.030	0.0
Alcohol abuse, n (%)	17 (2.7)	7 (1.6)	0.331	0.023	0.0
**Laboratory tests**					
WBC, k/uL	18.07 (12.44)	19.54 (20.90)	0.066	0.070	0.3
Hemoglobin, g/dL	9.54 (1.78)	9.60 (1.95)	0.505	0.031	0.1
Platelet, k/uL	191.86 (126.61)	184.37 (131.11)	0.234	0.057	0.3
Bilirubin, mg/dL	2.14 (4.30)	2.73 (4.73)	0.007	0.125	16.1
Serum creatinine, n (%)			0.003		0.1
< 1 mg/dL	196 (18.6)	102 ( 14.7)		0.108	
≥ 1, < 2 mg/dL	420 (39.8)	247 ( 35.7)		0.086	
≥ 2 mg/dL	439 (41.6)	343 ( 49.6)		0.160	
Urea nitrogen, n (%)			0.060		0.1
< 20 mg/dL	207 (19.6)	111 ( 16.0)		0.098	
≥ 20, < 40 mg/dL	409 (38.8)	258 ( 37.3)		0.031	
≥ 40 mg/dL	439 (41.6)	323 ( 46.7)		0.102	
Glucose, mg/dl	145.22 (47.17)	150.35 (58.20)	0.043	0.088	0.2
Lactate, mmol/L	3.45 (2.58)	4.68 (3.68)	< 0.001	0.334	4.4
Bicarbonate, mmol/L	19.09 (5.24)	17.86 (5.68)	< 0.001	0.217	0.3
INR	2.02 (1.66)	2.22 (1.71)	0.013	0.120	4.2
Potassium, mmol/L	4.72 (0.98)	4.88 (0.96)	0.001	0.168	0.1
Sodium, mmol/L	140.90 (5.97)	140.33 (6.39)	0.057	0.090	0.1
Urine output firstday, ml	1620.52 (1322.13)	1195.22(1060.30)	< 0.001	0.401	5.4
Mechanical ventilation firstday, n (%)	591 (56.0)	509 (73.6)	< 0.001	0.398	0.0
RRT firstday, n (%)	87 (8.2)	89 (12.9)	0.002	0.138	0.0
Vasopressin, n (%)	221 (20.9)	417 (60.3)	< 0.001	0.803	0.0
Dopamine, n (%)	171 (16.2)	140 (20.2)	0.037	0.100	0.0
Dobutamine, n (%)	49 (4.6)	65 (9.4)	< 0.001	0.163	0.0
Epinephrine, n (%)	7 (0.7)	50 (7.2)	< 0.001	0.253	0.0
Microorganism, n (%)	844 (80.0)	597 (86.3)	0.001	0.182	0.0
Initial NE dose, ug/kg.min	0.11 (0.09)	0.16 (0.31)	< 0.001	0.166	0.0
Initial PE dose, ug/kg.min	-	1.44 (1.25)	-	-	0.0

*NE* norepinephrine, *PE* phenylephrine, *MICU* medical intensive care unit, *SICU* surgical intensive care unit, *SOFA score* sequential organ failure assessment score, *APS III* acute physiology score III, *WBC* white blood cell, *INR* international normalized ratio, *RRT* renal replacement therapy, *SMD* standard mean difference

In the PSM cohort, 486 patients exposed to NE-PE were matched with 486 patients in the NE group at a 1:1 ratio. As indicated in Table 2, covariates for the matching cohorts were balanced between NE-PE and NE( all covariates SMD < 0.1).

**Table 2:** Baseline characteristics of NE and NE-PE group for matching cohort

Covariate	NE (*n* = 486)	NE-PE (*n* = 486)	*P*-value	SMD
Age, year	67.23 (15.95)	67.44 (15.40)	0.839	0.003
Gender, female, n (%)	220 (45.5)	217 (44.8)	0.897	0.031
Weight, kg	82.16 (26.92)	83.29 (24.94)	0.501	0.050
Ethnicity, n (%)			0.734	
White	360 (74.1)	354 (72.8)		0.016
Black	40 (8.2)	47 (9.7)		0.007
Other	86 (17.7)	85 (17.5)		0.024
Admission type, n (%)			0.976	
Emergency	468 (96.3)	467 (96.1)		0.090
Urgent	8 (1.6)	8 (1.6)		0.050
Elective	10 (2.1)	11 (2.3)		0.074
First careunit, n (%)			0.709	
MICU	353 (72.6)	354 (72.8)		0.004
SICU	57 (11.7)	50 (10.3)		0.024
Other	76 (15.6)	82 (16.9)		0.016
**Vital signs**				
Heart rate, bmp	95.59 (17.38)	95.48 (17.10)	0.927	0.034
Mean BP, mmHg	70.95 (7.55)	71.30 (8.28)	0.489	0.012
Respiratory rate, bmp	21.92 (4.73)	21.87 (4.61)	0.869	0.023
SpO2, %	96.58 (3.71)	96.48 (3.21)	0.655	0.027
Temperature, °C	36.84 (0.83)	36.86 (0.78)	0.725	0.022
SOFA score	9.16 (3.61)	9.11 (3.70)	0.833	0.025
APS III	70.88 (23.93)	71.29 (22.85)	0.789	0.027
Elixhauser comorbidity score	14.56 (8.29)	14.35 (8.59)	0.695	0.013
**Comorbidity**				
Congestive heart failure, n (%)	225 (46.5)	209 (43.2)	0.332	0.004
Cardiac arrhythmias, n (%)	249 (51.4)	239 (49.4)	0.563	0.010
Pulmonary circulation disorder, n (%)	48 (9.9)	47 (9.7)	1.000	0.003
Peripheral vascular disorder, n (%)	43 (8.9)	47 (9.7)	0.740	0.003
Neurological diseases, n (%)	56 (11.6)	66 (13.6)	0.383	0.024
Chronic pulmonary disease, n (%)	127 (26.2)	130 (26.9)	0.884	0.005
Hypertension, n (%)	247 (51.0)	246 (50.8)	1.000	0.010
Diabetes, n (%)	164 (33.9)	161 (33.3)	0.892	0.009
Renal failure, n (%)	126 (26.0)	122 (25.2)	0.825	0.010
Liver disease, n (%)	123 (25.4)	120 (24.8)	0.882	0.007
Metastatic cancer, n (%)	38 (7.9)	37 (7.6)	1.000	0.020
Solid tumor without metastasis, n (%)	19 (3.9)	17 (3.5)	0.865	0.020
Fluid and electrolyte disorders, n (%)	288 (59.5)	289 (59.7)	1.000	0.034
Drug abuse, n (%)	18 (3.7)	15 (3.1)	0.723	0.000
Alcohol abuse, n (%)	48 (9.9)	47 (9.7)	1.000	0.043
**Laboratory tests**				
WBC, k/uL	18.67 (11.95)	18.64 (11.99)	0.967	0.032
Hemoglobin, g/dL	9.60 (1.85)	9.64 (1.91)	0.745	0.017
Platelet, k/uL	189.31 (130.94)	190.31 (130.87)	0.905	0.043
Bilirubin, mg/dL	2.69 (5.50)	2.55 (4.65)	0.652	0.016
Serum creatinine, n (%)			0.733	
< 1 mg/dL	86 (17.7)	78 (16.0)		0.000
≥ 1, < 2 mg/dL	172 (35.4)	181 (37.2)		0.009
≥ 2 mg/dL	228 (46.9)	227 (46.7)		0.008
Urea nitrogen, n (%)			0.901	
< 20 mg/dL	79 (16.3)	84 (17.3)		0.017
≥ 20, < 40 mg/dL	182 (37.4)	182 (37.4)		0.019
≥ 40 mg/dL	225 (46.3)	220 (45.3)		0.031
Glucose, mg/dl	149.04 (49.06)	149.63 (51.74)	0.856	0.022
Lactate, mmol/L	4.11 (3.11)	4.22 (3.22)	0.563	0.038
Bicarbonate, mmol/L	18.21 (5.45)	18.38 (5.69)	0.628	0.022
INR	2.18 (1.94)	2.19 (1.84)	0.943	0.064
Potassium, mmol/L	4.83 (1.06)	4.86 (0.96)	0.690	0.023
Sodium, mmol/L	140.43 (5.93)	140.43 (6.82)	0.996	0.014
Urine output firstday, ml	1280.48 (1078.06)	1290.24 (1126.80)	0.891	0.018
Mechanical ventilation firstday, n (%)	341 (70.5)	333 (68.8)	0.625	0.026
RRT firstday, n (%)	52 (10.7)	53 (11.0)	1.000	0.009
Vasopressin, n (%)	212 (43.8)	225 (46.5)	0.438	0.068
Dopamine, n (%)	97 (20.0)	96 (19.8)	1.000	0.000
Dobutamine, n (%)	36 (7.4)	41 (8.5)	0.635	0.004
Epinephrine, n (%)	7 (1.4)	17 (3.5)	0.063	0.064
Microorganism, n (%)	417 (86.2)	407 (84.1)	0.416	0.012
Initial NE dose, ug/kg.min	0.12 (0.11)	0.13 (0.14)	0.168	0.035

*NE* norepinephrine, *PE* phenylephrine, *MICU* medical intensive care unit, *SICU* surgical intensive care unit, *SOFA score* sequential organ failure assessment score, *APSIII* acute physiology score III, *WBC* white blood cell, *INR* international normalized ratio, *RRT* renal replacement therapy, *SMD* standard mean difference

### Primary outcome

The hospital mortality rate was higher in patients who received NE-PE than in those who received NE (51.0% vs. 38.7%, *P* < 0.001) in the propensity score matching cohort, the univariate logistic regression analysis showed the NE-PE group has a higher hospital mortality rate compared to NE group (OR = 3.16, 95% CI = 2.59–3.88, *p* < 0.001), and the multivariate stepwise logistic regression analysis also demonstrated a higher hospital mortality rate in NE-PE versus NE (OR = 1.76, 95% CI = 1.36–2.28, *p* < 0.001). For the sensitivity analysis, as listed in Table 3, all two models yielded a similar result: patients in the NE-PE group had higher hospital and ICU mortalities compared to NE alone.

**Table 3:** The primary outcomes in four different models

Method	OR	95%CI	*P*-value
**Hospital mortality**			
Univariate logistic regression	3.16	2.59—3.88	< 0.001
Multivariate stepwise logistic regression	1.76	1.36—2.28	< 0.001
Propensity score matching	1.91	1.41—2.61	< 0.001
Doubly robust with all covariates	1.10	1.06—1.14	< 0.001
**ICU mortality**			
Univariate logistic regression	3.53	2.87—4.35	< 0.001
Multivariate stepwise logistic regression	1.94	1.49—2.54	< 0.001
Propensity score matching	1.97	1.45—2.75	< 0.001
Doubly robust with all covariates	1.11	1.07—1.15	< 0.001

*OR* odds ratio, *CI* confidence interval

### Secondary study outcomes in the PSM cohort

We assessed several secondary outcomes to explore potential reasons accounting for the higher mortality rate in the NE-PE group. A few differences were observed in secondary outcomes (Table 4). First, ICU LOS (12.18 vs 9.25 days, *p* < 0.001) and hospital LOS (17.35 vs 15.19 days, *p* = 0.019) were significantly longer in the NE-PE group. Second, the NE-PE group had longer durations of MV (160.26 vs 132.74 h, *p* = 0.042). The percentages of AKI at 48 h and 7 days after ICU admission did not differ between the groups.

**Table 4:** Secondary outcomes between NE and NE-PE group for matching cohort

Outcomes	NE (*n* = 486)	NE-PE (*n* = 486)	*P*-value
ICU LOS, day	9.25 (8.85)	12.18 (13.09)	< 0.001
Hospital LOS, day	15.19 (12.91)	17.35 (15.71)	0.019
AKI-48 h, n (%)	400 (82.3)	386 (79.4)	0.289
AKI-7 day, n (%)	439 (90.3)	433 (89.1)	0.598
IMV duration, hour	132.74 (187.79)	160.26 (231.41)	0.042

*NE* norepinephrine, *PE* phenylephrine, *ICU* intensive care unit, *LOS* length of stay, *AKI* acute kidney injury, *MV* mechanical ventilation

### Subgroup analyses for hospital mortality rate

**Fig. 2:**
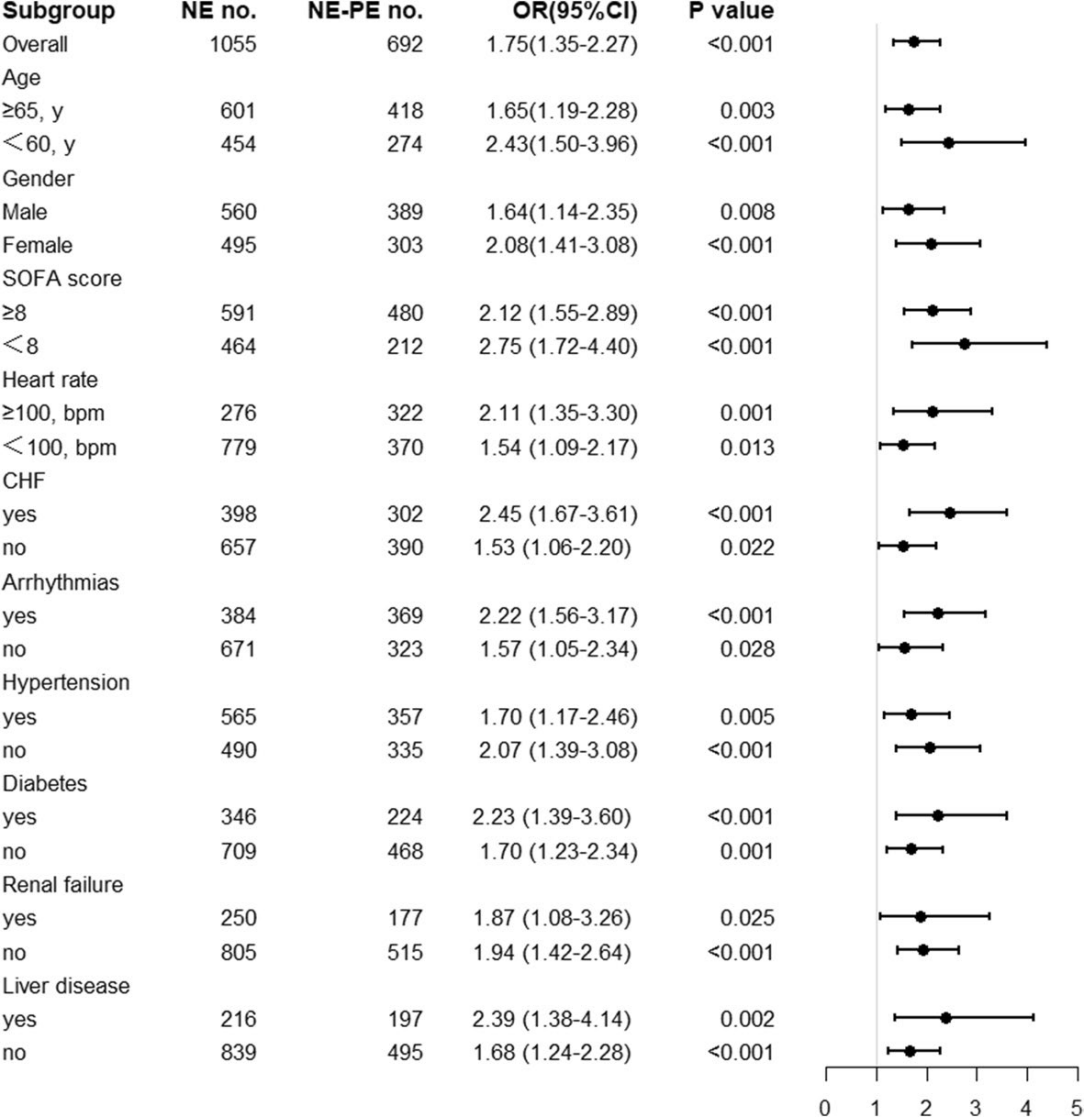
Forest plot of subgroup analysis of relationship between groups and mortality

As shown in Fig. 2, the hospital mortality rate did not differ significantly among the subgroups.

## Discussion

This retrospective, single-center cohort study found that NE-PE infusion did not lead to the same outcomes compared with NE in patients with septic shock. On the contrary, NE-PE administration increased the mortality risk, which was confirmed by the univariate and multivariate stepwise logistic regression model, PSM model, and doubly robust model. The total mortality rate in our study was 38.52%, which was consistent with an epidemiological survey by Bauer and Vincent et al. [1, 2]. In addition, NE-PE use was associated with longer ICU LOS, hospital LOS, and duration of mechanical ventilation, but there were no differences in the incidence rates of AKI at 48 h and 7 days after ICU admission.

The increased mortality of NE-PE may be attributable to several factors. As a selective α1-receptor agonist, PE increases systemic vascular resistance and arterial blood pressure while decreasing splanchnic perfusion and increasing arterial lactate in septic shock [27]. Splanchnic hypoperfusion may cause cytokine release into the systemic circulation in septic shock, which potentially leads to a vicious circle of inflammatory responses, culminating in multiple organ dysfunction syndromes [27, 28]. PE may also reduce cardiac output and cerebral oxygen saturation [14, 29]. Patients with septic shock often also experience cardiac dysfunction; PE leads to increased afterload, which offsets the benefits of cardiac output through arterial vasodilation caused by septic shock [30, 31]. NE has a lesser effect on cardiac output than PE due to its β1-adrenergic agonism. Moreover, a retrospective study found that patients who received PE had a greater decrease in HR over 24 h, which may have resulted in a decrease in cardiac output [15]. The above potential reasons are consistent with the SSC guidelines that do not recommend the use of PE [16]. The same results regarding the increased mortality in PE were also observed in studies by Hawn et al. [15] and Patel et al. [32].

Our study had the following specific strengths: we included a large population of 1, 747 patients with septic shock in a public database, and we performed multivariate stepwise logistic regression, PSM, doubly robust and subgroup analyses to improve the robustness of the results. However, the study also had several limitations. First, it had a retrospective design and was therefore subject to selection bias; although we used PSM and a multivariate model to control bias, other unknown confounding factors may remain. Second, we could not identify the specific clinical decision that would lead to the selection of PE for an individual patient, such as whether it was for a specific disease, during NE shortages, or based on the personal experience of the physician. Third, different therapeutic targets of NE, NE combined with PE, and other vasopressors in septic shock may leads to different endings, and this was not clear in our study. Fourth, although we have balanced the initial dose of NE by PSM, the subsequent infusion dose of NE and other vasopressors were not clear, and these differences may have an impact on outcomes. Fifth, the study was limited to a septic shock population from a single center, which makes it difficult to extrapolate our findings to different shock syndromes in critical care units.

## Conclusion

This study found that the infusion of NE combined with PE was associated with a higher hospital mortality rate in comparison with NE alone for patients with septic shock. This result reminds us that PE should be used with caution in patients with septic shock. The results need to be confirmed in multicenter prospective randomized clinical trials.

## Data Availability

The datasets presented in this study can be found in online repositories. The names of the repository/repositories and accession number(s) can be found below: The data were available on the MIMIC-III website at https://mimic.physionet.org/, http://dx.doi.org/10.13026/C2XW26.
